# 

**Published:** 1896-04

**Authors:** 


### Society Notes.

### Texas State Medical Association.

Preliminary announcement and program of the Twenty-eighth annual meeting, to be held at Fort Worth, Texas, Tuesday, Wednesday, Thursday and Friday, April 28th, 29th, 30th, May 1st, 1896:

COMMITTEES AND OFFICERS.

*Committee of Arrangements.*—Bacon Saunders, chairman; Frank Gray, F. D. Thompson, W. A. Adams, E. D. Capp, A. P. Brown.

*Reception Committee.*—J. T. Fields, chairman; R. B. Grammer, J. W. Irion, R. B. West, W. A. Duringer, H. C. Whitehead, James Anderson, J. M. Borders, S. L. Van Zandt, F. C. Todd, W. B. West. D. R. Fly, H. C. Stevens.

OFFICERS.

President, P. C. Coleman, Colorado.
First Vice President, J. F. Wagley, Cleburne.
Second Vice President, N. B. Kennedy, Hillsboro.
Third Vice President, T. B. Bass, Terrell.
Secretary, H. A. West, Galveston.
Treasurer, J. Larendon, Houston.
Orator, Irwin Pope, Tyler.

OFFICERS OF SECTIONS.

I. General Medicine, W. L. York, Decatur, chairman; R. B. Grammer, Fort Worth, secretary.

II. Obstetrics and Diseases of Children, J. J. Williamson, Cleburne, chairman; C. M. Alexander, Coleman, secretary.

III. Surgery, M. D. Knox, Hillsboro, chairman; O. L. Williams, Dallas, secretary.

IV. Medical Jurisprudence, etc., T. J. Bennet, Austin, chairman; Sam Cunningham, Taylor, secretary.

V. Gynecology, T. J. Bell, Tyler, chairman; J. F. Letcher, Dallas, Secretary.

VI. State Medicine, etc., R. Rutherford, Houston, chairman; I. N. Suttle, Corsicana, secretary.

VII. Ophthalmology, etc., J. O. McReynolds, Dallas, chairman; H. L. Hilgartner, Austin, secretary.

VIII. Dermatology, etc., Geo. H. Lee, Galveston, chairman; R. W. Knox, Houston, secretary.

IX. Microscopy, etc., J. E. Brown, McGregor, chairman; Wm. Gammon, Galveston, secretary.

TRANSPORTATION.

The following railroads offer a rate of one and a third fare on the certificate plan: Fort Worth and Denver; Chicago, Rock Island and Texas; G. C. & S. F.; St. Louis and Southwestern; T. & P.; Fort Worth and Rio Grande; H. & T. C.; Transcontinental; M. K. & T.

Wm. Doherty, No. 403 Main st., of the G. C. & S. F., has been appointed joint agent to stamp certificates. Passengers should secure receipt from agent at point of departure, which must be signed by Dr. H. A. West, secretary, and stamped as above.

Certificates will not be executed by the joint agent for reduced rates returning, if presented more than one day after adjournment of the meeting.

Delegates are requested to present their certificates upon the first day of meeting.

### HOTEL RATES.

Worth, $2.50 per day, ordinary rate, $3.00.

Delaware, $2.50 per day, ordinary rate, $3.00.

Mansion, $1.75 per day, ordinary rate, $2.00.

### PRELIMINARY PROGRAM.

The session will begin Tuesday morning, April 28th, at 11 o'clock. Members are requested to assembly early, in order to register, as far as possible, before the opening. The secretary, treasurer, and members of the reception committee will be present to attend to registration. Applicants for membership will sign blank form, giving postoffice address and county. Each application must have the signature of two members, and be accompanied by the initiation fee of $5.00, and dues for one year, $5.00. The diploma, or other documentary evidence of graduation, must be furnished to the judicial council. Applicants will receive badges when they exhibit the treasurer's receipt. Members will receive badges after they register, and are urgently requested to register promptly. The badge is an evidence of membership, and will not be given to non-members.

As the titles of papers appearing in this program were received in time to be arranged in proper order, they will take precedence of those offered subsequently.

### ORDER OF EXERCISES.

Tuesday, 11 a. m.

Call to order by chairman committee of arrangements, Dr. Bacon Saunders.

Invocation.

Welcome by mayor of Fort Worth.

Welcome by representatives of the medical profession of Fort Worth.

Response to welcome addresses, P. C. Coleman, president.

Roll call.

Reading minutes of previous session.

Secretary's Annual Report.—H. A. West.

Treasurer's Annual Report.—J. Larendon.

President's Annual Message and recommendations—.P. C. Coleman.

SECTIONS.—GENERAL MEDICINE.

I. Report of Chairman.—W. L. York, Decatur.

II. Newer Methods of Treatment of Nervous and Mental Disease.—Frederick Peterson, New York.

III. Some Settled Questions in Regard to Diphtheria.—H. A. West, Galveston.

IV. Diphtheritis and Membranous Croup, with Practical Demonstrations.—S. T. Turner, El Paso.

V. Diphtheria and Associated Diseases.—E. W. Capps, Fort Worth.

VI. Diphtheria.—S. S. Grenewell, Cleburne.

VII. Diphtheria and Psuedo-Diphtheria, their Differential Diagnosis by the Practitioner.—C. O. Mathews, Terrell.

VIII. Influence of Climatic Conditions and Weather Changes on the Functions of the Skin.—Isaac M. Cline, Galveston.

IX. Notes on the Action of Apolysin.—David Cerna, Galveston.

X. Typhoid Fever.—W. B. McKnight, Mansfield.

XI. The Treatment of Appendicitis Considered from a General Standpoint.—A. H. Schenk, Kenney.

XII. Hæmatemesis, Report of Case.—W. R. Blailock, McGregor.

XIII. The Treatment of Pneumonia.—R. L. Gilbert, Oak Cliff. Discussion opened by Dr. Stout, Dallas.

XIV. The Prevailing Diseases of East Texas, and the Changes Thereof During the Past Thirty Years.—H. L. Tate, Lindale.

OBSTETRICS AND DISEASES OF CHILDREN.

I. Report of Chairman.—J. J. Williamson, Cleburne.

II. External vs. Internal Examination in Obstetrics.—W. M. Powell, Albany.

III. Puerperal Septicæmia.—B. H. Vaughan, Hillsboro.

IV. Puerperal Peritonitis.—R. B. McKinney, Memphis, Tenn.

V. Report of a Case of Cæsarian Section.—J. E. Gilcreest, Gainesville.

VI. An Encephalocele.—A. P. Brown, Fort Worth. Discussion opened by R. M. Swearingen, Austin.

VII. Several Cases of Puerperal Septicaemia from a Case of Facial Erysipelas.—W. M. Yates, Grandview. Discussion opened by A. M. Douglas.

VIII. Scarlatina.—G. C. Head, Grandview.

SURGERY.

I. Report of Chairman.—M. D. Knox, Hillsboro.

II. Some of the Later Methods of Treating Tubercular Joints and Correcting Deformities Resulting from Infantile Paralysis.—M. M. Edmonson, Dallas. Discussion opened by J. H. Smart, Dallas.

III. Tuberculosis of Bone, Especially that of Knee Joint.—W. P. Alexander, Cleburne. Discussion opened by W. T. Baird, Dallas.

IV. Some Cases of Osteomyelitis.—W. R. Blailock, McGregor.

V. Importance of Rest and Extension in Traumatisms of the Cervical Vertabræ.—Geo. H. Lee, Galveston.

VI. Treatment of Compound Depressed Fractures of the Skull.—A. C. Scott, Temple. Discussion opened by C. A. Smith, Tyler.

VII. Early Extirpation of an Intra-Mammary—Adeno-Sarcoma.—F. E. Haynes, Abilene. Discussion opened by R. R. Walker, Paris.

VIII. Carbuncle with Grave Complications.—O. L. Williams, Dallas. Discussion opened by D. Dupree, Dallas.

IX. Report of a Case of Encephaloid Cancer of the Kidney.—W. J. Lane, Dallas. Discussion opened by Allen J. Smith, Galveston.

X. Asepsis in Surgery.—Z. T. Bundy, Milford. Discussion opened by T. F. Oates, Mexia.

XI. Habitual Constipation a Surgical Disease.—W. T. Baird, Dallas. Discussion opened by Sam R. Burroughs, Raymond.

XII. The Modern Method of Treating Sprained Ankles—J. E. Gilchreest, Gainesville. Discussion opened by A. P. Brown, Fort Worth.

XIII. The Surgical Anatomy of the Vermiform Appendix and Caecum.—W. Keiller, Galveston.

XIV. Some Remarks on the Surgery of the Kidney.—J. E. Thompson, Galveston.

XV. Chloroform Anæstheia.—H. P. Cooke, Galveston.

XVI. Chronic Posterior Urethritis.—J. J. Bush, Pecos. Discussion opened by Dr. W. A. Adams, Fort Worth.

XVII. Two Cases of Cholecystotomy.—A. W. Fly, Galveston.

### MEDICAL JURISPRUDENCE, ETC.

I. Report of the Chairman.—T. J. Bennett, Austin.

II. A Plea for Reform in Criminal Jurisprudence.—F. E. Daniel, Austin.

### GYNECOLOGY.

I. Report of Chairman.—T. J. Bell, Tyler.

II. Immediate Repair of Lacerations of the Perineum.—J. M. Richmond, Edna.

III. Tubal Pregnancy and its Termination.—Joseph Price, Philadelphia.

IV. Urethral Caruncle.—B. F. Brittain, Arlington.

V. Some Mistakes of Surgical Gynecology.—Thos. A. Stoddark, Pueblo, Col.

VI.—Subject not Announced.—J. T. Jelks, Hot Springs, Ark.

VII. Appendicitis in its Relations to Gynecology and Obstetrics.—By Dr. Paul F. Munde, of New York.

VIII. Post Operative Complications about the Abdominal Incision.—By Dr. Joseph Price, of Philadelphia.

IX. Subject Unannounced.—By Dr. Richard Douglass, of Nashville, Tenn.

X. A Case of General Peritonitis, due to Suppressed Menstruation, in a 12-Year-Old Girl.—By Dr. Joe D. Becton, of McKinney, Tex.

XI. Conservatism as Applied to Gynecology.—By Dr. R. R. Walker, of Paris, Texas.

### STATE MEDICINE AND PUBLIC HYGIENE.

I. Report of Chairman.—R. Rutherford, Houston.

II. How to Dispose of Liquid Waste in Towns Which Have Water Works but no Sewers.—Wm. M. Yandell, El Paso.

III. State Care of Insane Epileptics, Inebriates and Habitues of Narcotics.—F. S. White, Terrell.

IV. Medical Education, its Defects and Perversions.—Jos. A. Mullen, Houston.

### OPHTHALMOLOGY, ETC.

I. Report of Chairman.—John O. McReynolds, Dallas.

II. Methods of Treatment of Secondary Cataract.—Henry Power, London, England.

III. The Eye in Relation to Health.—H. L. Hilgartner, Austin.

IV. The Relation of Ophthalmology to General Medicine.—Harry Friedenwald, Baltimore, Md.

V. Eye Surgery by the General Practitioner.—E. J. Neathery, Van Alstyne.

VI. Eye Troubles Commonly Arising in General Practice.—G. W. Grove, Kansas City, Mo.

VII. Report of Cases.—R. E. Moss, San Antonio.

VIII. The Nature of Glaucoma and the Method of Treating the Disease by Sclerotomy.—X. Galezowski, Paris, France.

IX. Ocular Effects of La Grippe. Report of a Case.—R. L. Miller, Sherman.

X. Relation existing Between Diseases of the Eye and Brain.—R. F. LeMond, Denver, Colo.

XI. Epilepsy as a Result of Nasal Obstruction.—Frank C. Todd, Fort Worth.

XII. Cataract, Diagnosis and Treatment.—R. H. Chilton Dallas.

XIII. Functional Impairment of the Auditory Center as Result of Catarrhal Deafness.—Joseph A. Mullen, Houston.

XIV. Some Notes on Laryngology.—Frank W. Boyd, San Antonio.

XV. Some Observations on the Examination and Treatment of Strabismus.—Vard H. Hulen, Galveston.

XVI. The Cure of Deviations of the Nasal Septum.—Vard H. Hulen, Galveston.

### DERMATOLOGY, ETC.

I. Report of Chairman.—George H. Lee, Galveston.

II. Title to be announced. George Henry Fox, New York.

III. Title to be announced—A. Van Harlingen, Philadelphia.

IV. Erythema Multiform Following Circumcision.—R. W. Knox, Houston.

V. A Peculiar Rash Accompanying Malarial Fever.—Allen J. Smith and Wm. Gammon, Galveston.

### MICROSCOPY AND PATHOLOGY.

I. Report of Chairman.—J. E. Brown, McGregor.

II. Pathology and Diagnosis of Pleural Effusions.—W. F. Starley, Jr., Galveston.

The indications are favorable for a large attendance at Fort Worth. The Committee of Arrangements are making preparation in accordance with their well known energy and hospitality.

The above list of papers, while incomplete from the failure of officers to report, is, nevertheless of sufficient scope to guarantee an interesting and instructive session.

The committee appointed at the last session to devise ways and means for the good of the association and to formulate a bill to regulate the practice of medicine in Texas, met in Dallas, March 28th. All members were present. The report is ready, and its consideration will constitute one of the most important subjects ever brought before the association. The attendance of all regular and reputable physicians in the State is earnestly solicited.

H. A. West, Secretary.

---

The order of the titles listed above was hastily prepared, and is subject to revision by the Secretary.

### American Medical Association.—Section on Neurology and Medical Jurisprudence.

The next annual meeting will be held at Atlanta, Ga., May 5th to 8th, 1896.

ANNOUNCEMENT.

The work of the Section on Neurology and Medical Jurisprudence at the coming meeting of the American Medical Association at Atlanta, promises to be of unusual interest. The desire has been quite generally expressed to have discussions upon the following topics:

The Etiology of Insanity.

Expert Medical Testimony in Disputed Mental Cases.

Medical Aspects of Crime.

It is believed that these topics have a special medical interest at this time, and papers dealing with any phase of these subjects will be very welcome. An urgent invitation is extended to all members, whether they are expecting to be in attendance or not, to present papers and records of cases that have a bearing upon the topics mentioned, or upon any other Neurological or Medico-Legal subject.

Any persons who expect to contribute papers or reports to this section will greatly facilitate the business of the section by writing at once to the chairman,

T. D. CROTHERS, M. D., Hartford, Conn.

Or to the secretary,

W. J. HERDMAN, M. D., Ann Arbor, Mich.

Dr. D. R. Brower, of Chicago, will open the discussion on the Medical Aspect of Crime. Dr. F. E. Daniel, of Austin, Texas, has been requested to follow him, and others will participate in the discussion. The subject is growing in interest and attention.

